# Comparative Analysis of Diagnostic Accuracy of OPG and CBCT in the Evaluation of Impacted Mandibular third Molar Proximity to the Mandibular Canal

**DOI:** 10.2174/0115734056307388260321064549

**Published:** 2026-04-03

**Authors:** Bhavana Sujanamulk, Ahmed A Almeshari, Nayanala Venkata Anusha, Chaganti Ashok, Bharani Krishna Takkella, Rohith Vadlamani

**Affiliations:** 1 Department of Oral & Maxillofacial Surgery and Diagnostic Sciences, Division of Oral Medicine & Radiology, Faculty of Dentistry, Najran University, Najran, Kingdom of Saudi Arabia; 2 Department of Oral Medicine & Radiology, Drs. Sudha and Nageswararao Siddhartha Institute of Dental Sciences, Dr. N.T.R University of Health Sciences, Vijayawada, Andhra Pradesh, India; 3 Department of Conservative Dentistry and Endodontics, Nimra Dental College, Dr.N.T.R University of Health Sciences, Jupudi, Andhra Pradesh, India; 4 General Dentist, Terre Haute, Indiana, United States of America

**Keywords:** CBCT (cone beam computed tomography), Impacted mandibular molar, Rood and Shehab’s radiographic signs, IAN (inferior alveolar nerve), MC (mandibular canal), OPG, Alveolar nerve damage

## Abstract

**Introduction:**

Mandibular third molars are the most frequently impacted teeth. The proximity of impacted third molars to the inferior alveolar canal requires particular consideration during extraction. Hence, we aimed to compare the diagnostic accuracy of CBCT and OPG using radiographic signs of Rood and Shebab’s and find out the risk of mandibular nerve injury.

**Methods:**

A prospective study was conducted in 50 subjects. All patients underwent digital OPG evaluation based on Winter’s classification of impacted teeth and Rood and Shehab’s classification of seven radiographic signs. Cases with any positive finding were examined using CBCT to assess the positioning of the mandibular canal and alveolar ridge.

**Results:**

Horizontal and mesioangular impactions were at higher risk of inferior alveolar nerve damage (*p*< 0.01 and *p* < 0.05) along with lingual position of canal (*p*<0.001), Darkening of roots, interruption of the mandibular canal (*p*<0.001), and diversion of the mandibular canal (*p*< 0.04) were associated with a higher risk of damage during extractions.

**Discussion:**

The current study confirmed that horizontal and mesioangular impactions showed a significant association with the absence of cortication between the mandibular canal and third molar roots, indicating a high risk of nerve injury, similar to other studies. Also, CBCT has been proven to be the best imaging modality owing to its high diagnostic accuracy.

**Conclusion:**

In the current study, CBCT was considered the gold standard, and diagnostic accuracy was higher than OPG. There was fair agreement about the mesioangular and horizontal impaction and their proximity to the mandibular nerve.

## 1 INTRODUCTION

An impacted tooth fails to erupt into the dental arch within the expected timeframe. This condition is often associated with complications, including pericoronitis, caries on the distal surface of the second molar, pain, swelling, bleeding, infection, external root resorption, and odontogenic cysts and tumors, most of which are temporary in nature [1]. The most significant complication involves partial or complete nerve damage, which is invariably iatrogenic and may result in paraesthesia of the chin and lower lip. The proximity of impacted third molars to the inferior alveolar canal (IAC) requires particular attention due to the elevated risk of nerve injury during surgical procedures [2]. Maxillofacial radiology is a branch that deals with the diagnosis of orofacial pathologies through various imaging modalities, including Radiovisography (RVG), digital orthopantomogram (OPG), and cone beam computed tomography (CBCT). While neurological complications resulting in paresthesia occur in 0.5–1% of cases, approximately 5–7% result in permanent nerve damage [3]. The risk increases significantly with proximity to the inferior alveolar nerve (IAN), highlighting the importance of understanding the relationship between impacted teeth and the IAN canal. Several classification systems have been proposed to reduce complications, with Winter’s and Pell and Gregory’s systems being widely adopted globally [4, 5].

A preoperative radiograph is mandatory during treatment planning for patients with impacted teeth. Dental OPG is the most commonly used radiographic method for assessing the jaws in cases of impacted third molars. OPGs provide several advantages to clinicians, including broad coverage of the oral and maxillofacial structures, detailed visualization of pathological lesions, low cost, and low radiation dose [6]. However, they also have limitations such as low image resolution and shape distortion. The major drawback is the inability to assess the risk of damage to the IAC and its relation to other vital structures, as it is a two-dimensional radiograph. Certain specific signs in OPG, such as darkening of roots, deflection of roots, bifid root apex, root narrowing, canal narrowing, canal diversion, and canal interruption, are used according to Rood and Shehab’s radiographic criteria to assess the risk of injury to the IAN [7].

Cone beam computed tomography has revolutionized diagnostic radiology through its ability to generate three-dimensional volumetric data for evaluating the oral and maxillofacial region [8]. It offers numerous advantages, including enhanced spatial resolution, accuracy, and detailed micro-imaging of specific regions, while maintaining lower costs and reduced radiation exposure compared to conventional CT procedures [9]. CBCT’s primary advantage lies in its ability to produce three-dimensional views of impacted mandibular third molars in relation to anatomical landmarks such as buccolingual cortical plates and the IAC, significantly reducing the risk of potential injuries [10]. Moreover, with recent advancements, the AI-based detection and evaluation of third molars employing the artificial neural networks, the convolutional neural networks (CNNs), and Deep learning models, have been effective in object detection, classification, and segmentation for the evaluation of impacted third molars from the panoramic radiographs and CBCT images. This development could help in obtaining diagnostic accuracy and enhancing the dental practice. However, AI could never replace dentists or radiologists since it may lead to misdiagnosis or overdiagnosis, resulting in false interpretations. Orhan *et al*. [11] evaluated the use of AI for detecting impacted third molars and their relation with anatomic structures and found high accuracy, where excellent agreement could be seen between the Human observation and AI detection. Likewise, in another study, they determined the accuracy of AI in detecting the position between the mandibular third molar and the inferior alveolar nerve on panoramic radiography and concluded that buccolingual positioning of IAN can be easily detected with AI, which could help in decision-making for the oral surgeons. While numerous studies have compared Rood and Shehab’s radiographic signs on OPG with CBCT to identify relationships between impacted third molars and the IAC, no study has provided definitive validation.Hence, to disprove the null hypotheses, the current study aimed to compare and evaluate the diagnostic accuracy and reliability of OPG and CBCT in assessing the relationship between the roots of impacted mandibular third molars and the mandibular canal with the objective of determining the imaging modality that provides superior anatomical delineation for preoperative risk assessment.

## 2 MATERIALS AND METHODS

### 2.1 Study Design and Sampling 

The current study was conducted at Drs. Sudha & Nageswara Rao Siddhartha Institute of Dental Sciences, Chinnaoutpalli, Andhra Pradesh, India. The study included 50 patients (25 males and 25 females) who visited our outpatient department with the chief complaint of pain in relation to the right or left side of the jaw and were diagnosed with impacted lower mandibular third molars aged 18–40 years during the time period of November 2024 to March 2025, and were selected through a simple random sampling method. The study adhered to the Sex and Gender Equity in Research (SAGER) guidelines to ensure proper inclusion and transparency in reporting of sex-related data related to our research. The study was approved by the Institutional Ethical Committee of Dr. Sudha & Nageswara Rao Dental College bearing (IEC No:11/2024), India. Both verbal and written informed consent were obtained from participating patients before the commencement of our study. A detailed case proforma was collected and examined for clinical symptoms pathognomonic of impaction. The study excluded subjects with chronic cold, sinusitis, facial deformities, previous maxillofacial trauma, head and neck surgeries, malignancies, systemic diseases, craniofacial anomalies, cysts, and tumors affecting the oral and maxillofacial region.

**Fig. (1):**
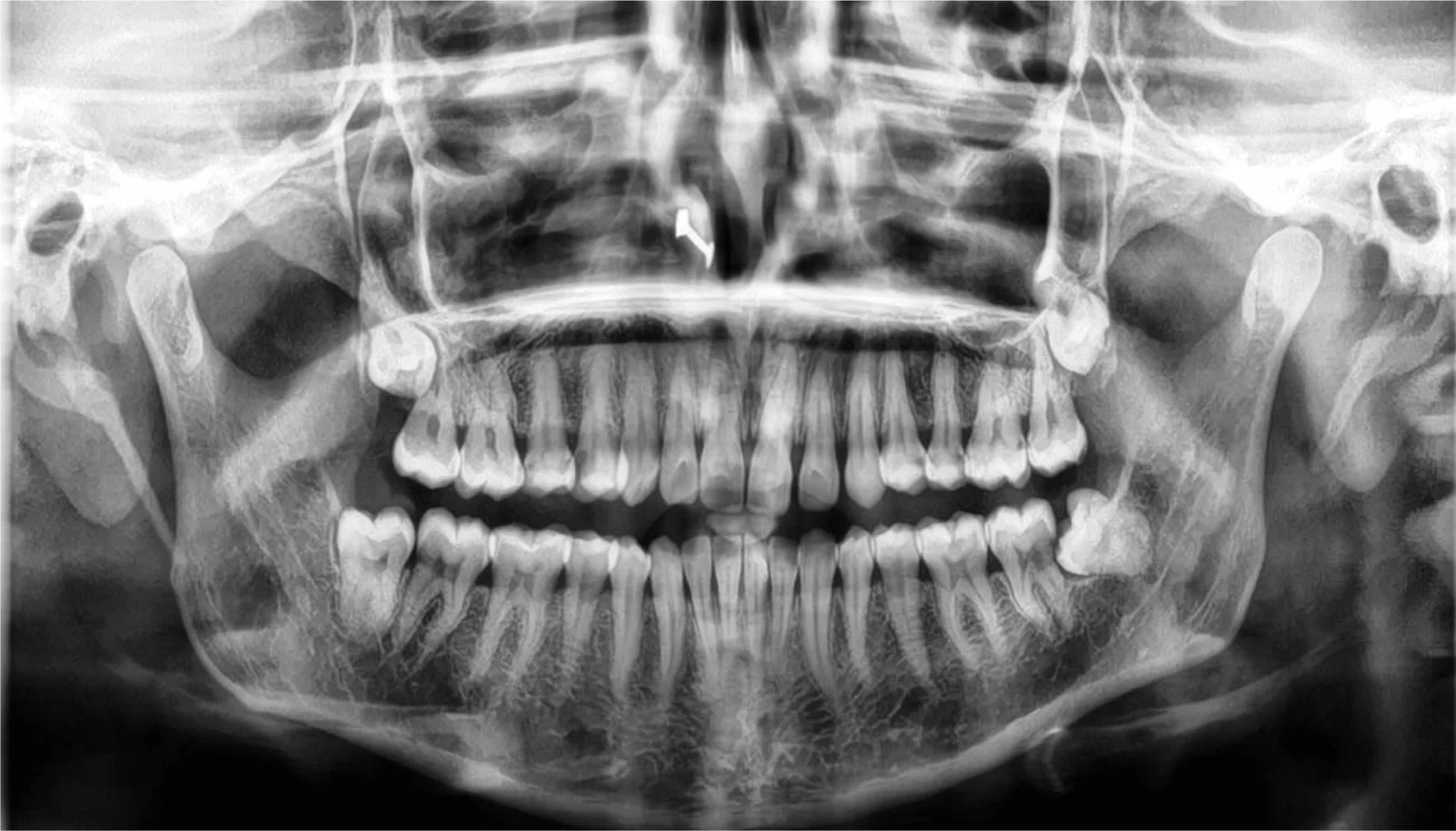
OPG of an impacted mandibular third molar.

**Fig. (2):**
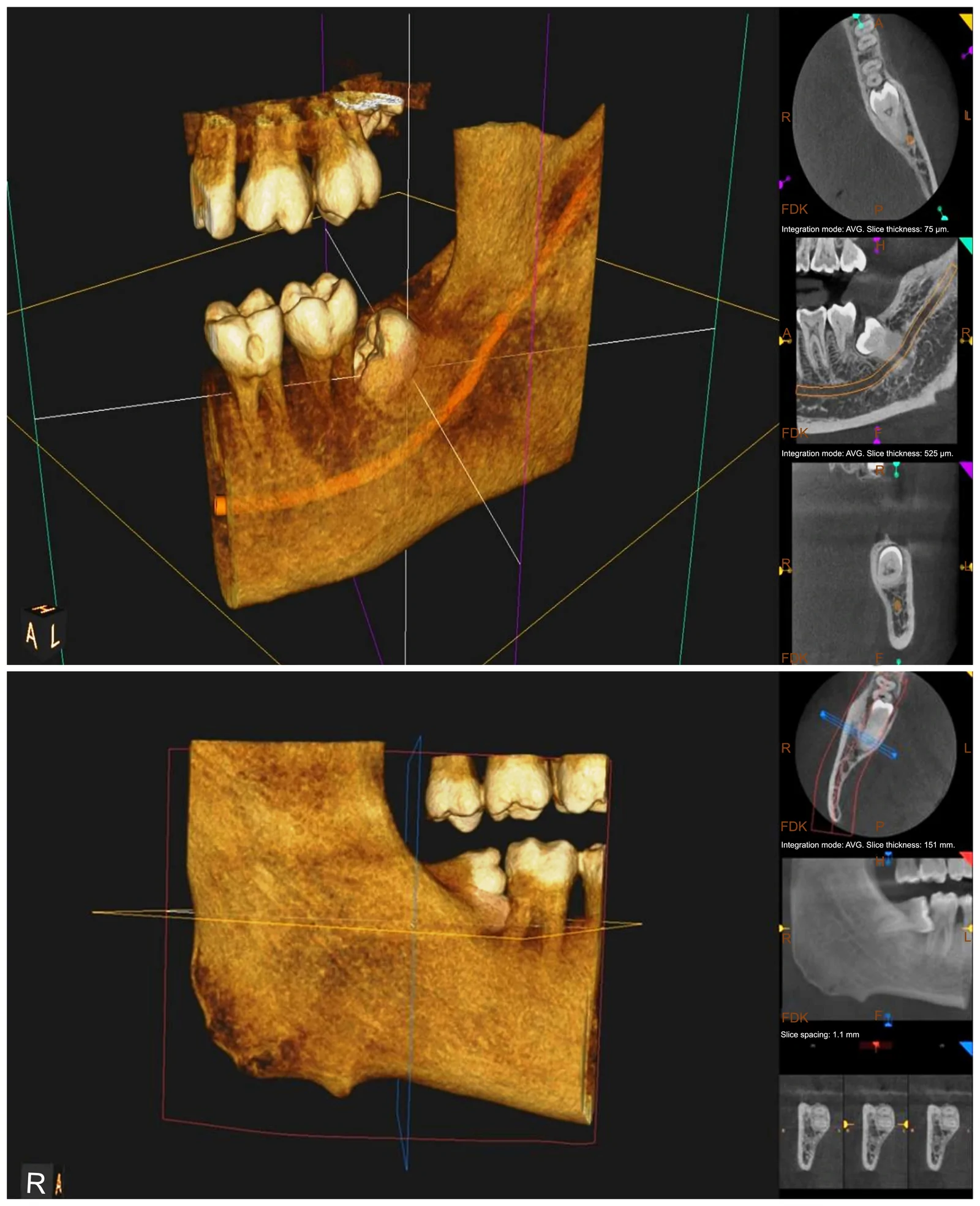
CBCT views of impacted mandibular third molar.

A diagnostic panoramic radiograph was obtained for each patient using a CS 9600 machine with specifications of 73 kVp, 8 mA, 12.3 s, with an equivalent dose of 122 mGy, operating at a filtration of 1.5 mm Al. The classification of impacted teeth followed Winter’s classification, mesioangular, distoangular, horizontal, and vertical, based on radiological correlation (Fig. **1**) [4]. These radiographs were evaluated for Rood and Shehab’s radiographic signs in OPG, including darkening of roots, deflection of roots, narrowing of roots, bifid root apex, interruption of canal, division of canal, narrowing of canal by two experienced oral and maxillofacial radiologists, who also examined the presence or absence of cortication along with position of canal to the alveolar ridge [7,12,13]. Subsequently, patients underwent CBCT scans of the mandible to observe the impacted mandibular third molar (Fig. **2**). Patient scans were obtained using a CS 9600 with CS 3D imaging software, operating at 120 kVp, 6.3 mA, 20.0 s, with an equivalent dose of 814 mGy and a filtration of 0.7 mm Cu, using a field of view of 12 cm × 5 cm. For scanning, patients stood in the spherical gantry housing the X-ray source and sensors, with height adjusted according to patient dimensions. Distorted images were excluded from the study. Images were analysed in DICOM format. CBCT image evaluation was conducted in a quiet environment with dim lighting. Images were inspected on a 1366 × 768 resolution Dell Inspiron laptop (i7 processor, 2.60 GHz, 8 GB RAM), with assessments analysed at high magnification in axial, coronal, and sagittal sections, using a standardized 150 mm slice thickness.

### 2.2 Comparison of OPG and CBCT 

**Fig. (3):**
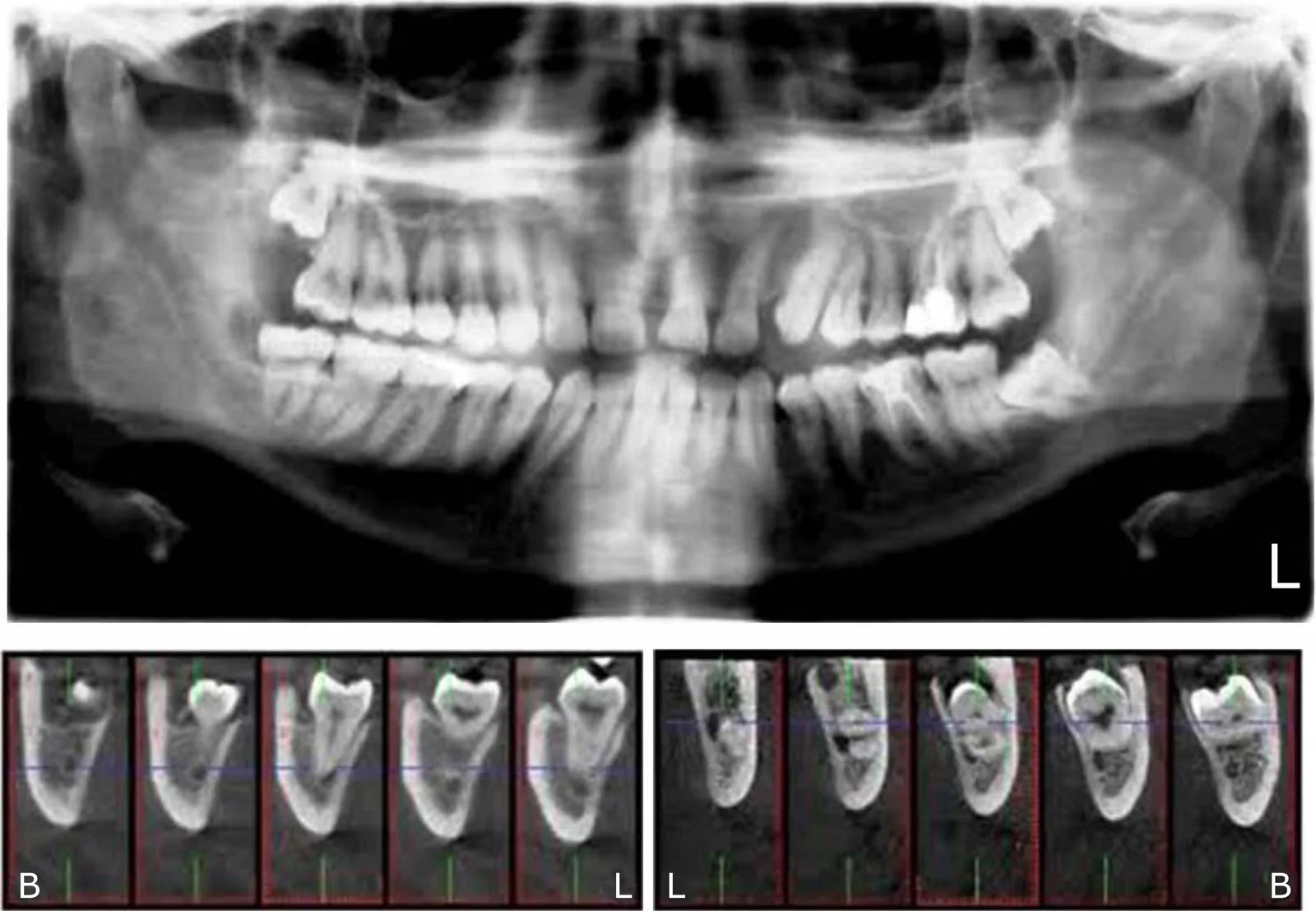
Comparison of OPG and CBCT images for assessment of cortication.

**Fig. (4):**
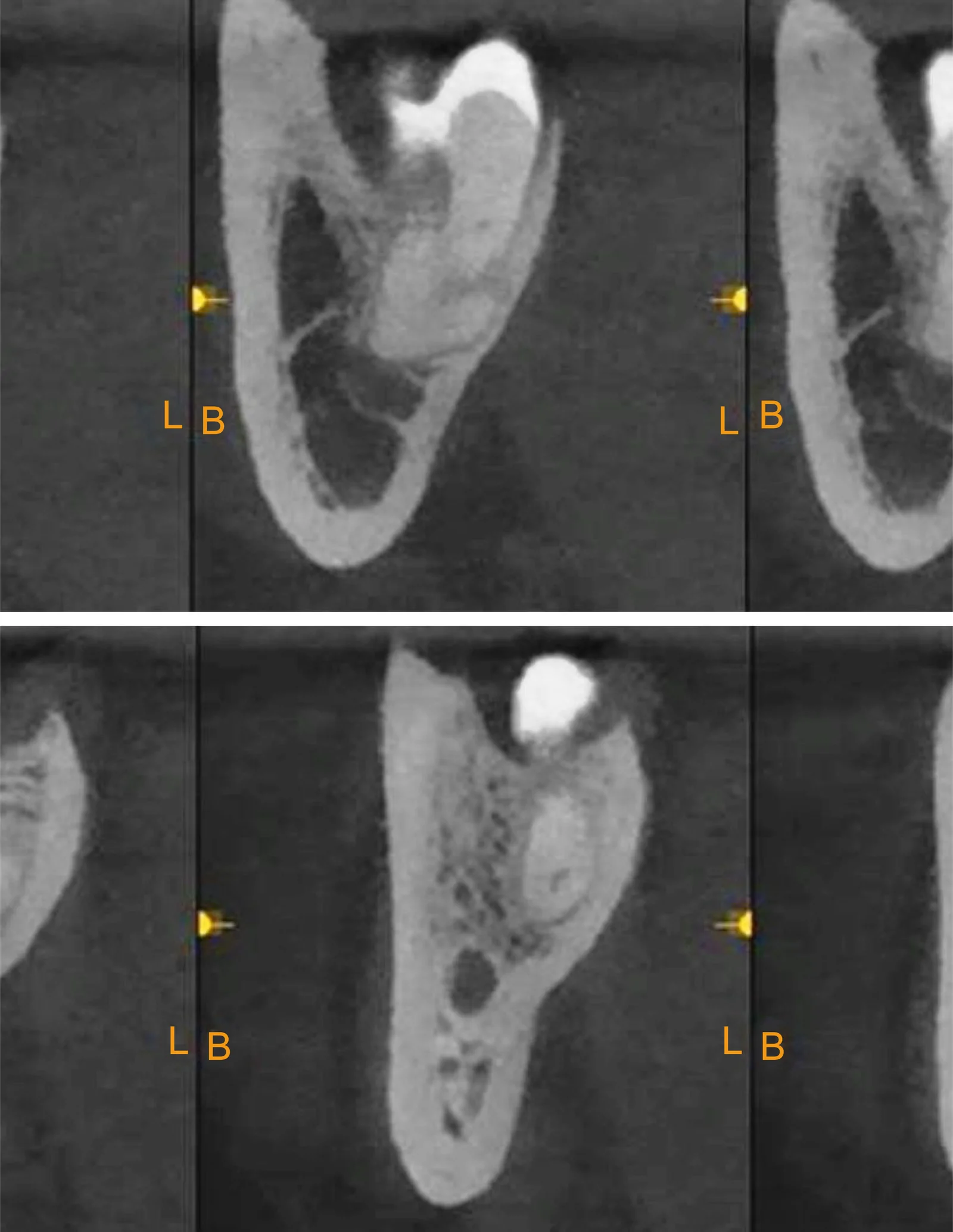
(**a**) Loss of cortication (**b**) presence of cortication seen in sagittal section.

Following initial OPG evaluation, each case was sent for CBCT analysis to determine the relationship between the impacted mandibular third molar and the IAC, assessed according to Rood and Shehab’s radiographic signs in OPG, such as darkening of roots, deflection of roots, narrowing of roots, bifid root apex, interruption of canal, division of canal, and narrowing of canal were compared and analysed (Fig. **3**). Later on, CBCT images were examined for the presence of cortical bone between the IAC and the impacted third molar while evaluating its position with the ridge. Absence of clear differentiation between these structures indicated an association between them, suggesting a potential risk of paraesthesia after extraction (Fig. **4a**, **b**). Hence, we followed STARD guidelines 2015 to evaluate the diagnostic accuracy of OPG and CBCT for evaluating our parameters.

## 3 RESULTS

### 3.1 Statistical Analysis 

The data collected were entered in to MS Excel sheet and were analysed by using SPSS Version 23.0(SPSS, INC., Chicago, IL, USA). Kappa statistics were calculated, and the value was 0.38, which was with fair agreement. Descriptive statistics were performed, and results for each categorical measurement were given as numbers. The obtained data were assessed using the Chi-square test, and *p*≤0.05 was considered statistically significant, and *p*≤0.001 was considered highly significant. Diagnostic accuracy tests, such as sensitivity, specificity, PPV, NPV, and accuracy, were calculated and included in the table.

Sensitivity = TP (True Positive)/TP (True Positive) +FN (False Negative)

Specificity = TN (True Negative)/ TN (True Negative) +FP (False Positive)

PPV = TP (True Positive)/TP (True Positive) + FP (False Positive)

NPV = TN (True Negative)/ TN (True Negative) + FN (False Negative)

Accuracy= TP (True Positive) + TN (True Negative)/ TP (True Positive) + FP (False Positive) + TN (True Negative) + FN (False Negative)

**Fig. (5):**
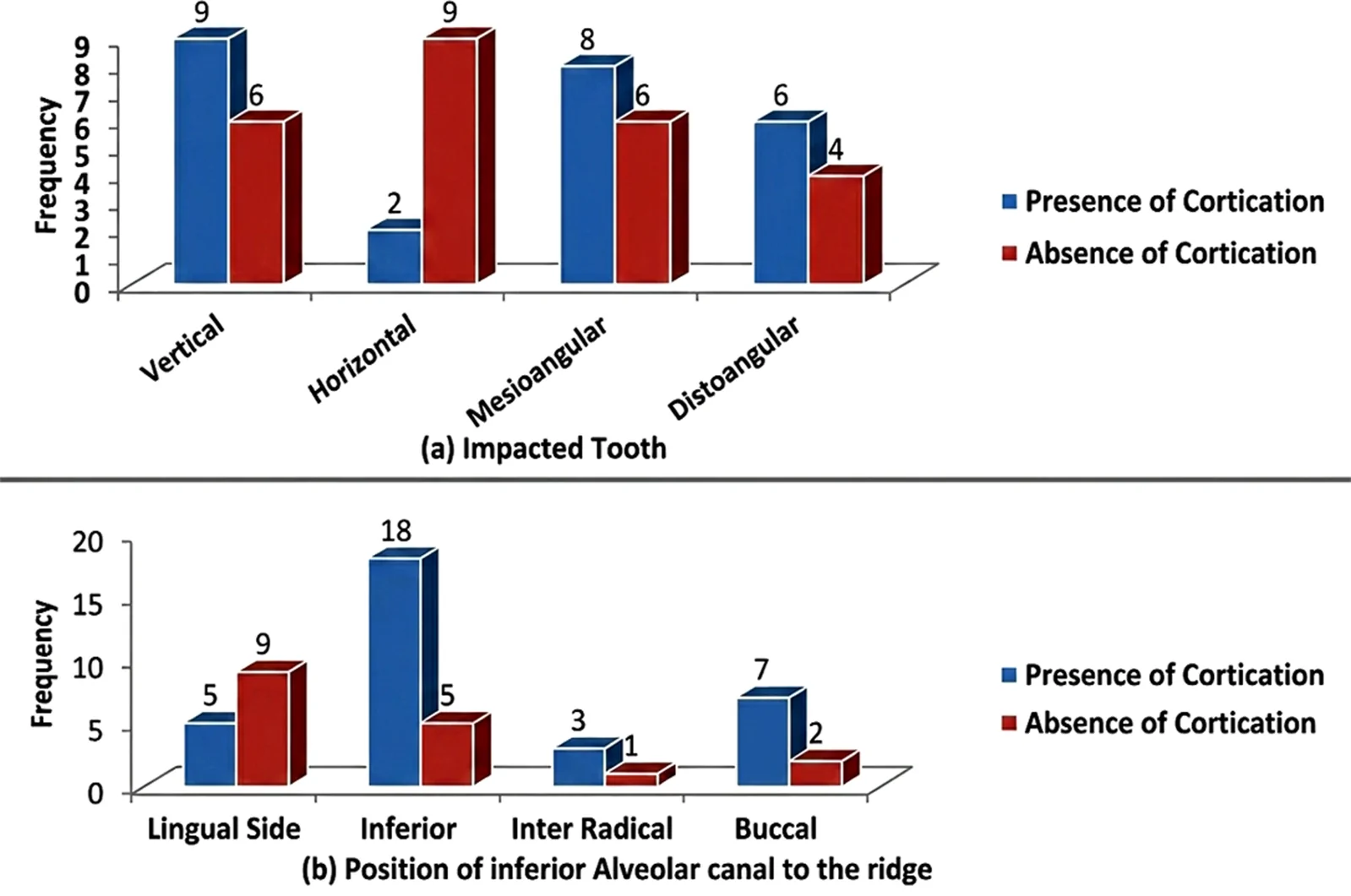
(**a**) Graph representing the assessment of cortication in different types of impacted mandibular third molars, (**b**) Graph representing the position of the inferior alveolar canal.

Table **1** the tooth positioning of impacted mandibular molars was assessed based on the presence or absence of cortication in CBCT. Presence of cortication is seen in 9 cases of vertical impaction, 2 cases of horizontal impaction, and 8 cases of mesioangular impaction, along with 6 cases of distoangular impactions respectively. However, horizontal and mesioangular impaction showed significant results with (*p*<0.01) for horizontal and (*p*<0.05) for mesioangular impaction, respectively. (Fig. **5a**).

Table **2** the position of the inferior canal with the presence or absence of cortication was assessed in CBCT. Among these, the inferior position of IAC had more cortication recording 18 cases, followed by buccal, which included 7 cases, then 5 cases lingual and interradicular with 3 cases. Highly significant results were obtained with regard to the lingual position of IAC (*p*<0.001). (Fig. **5b**).

**Fig. (6):**
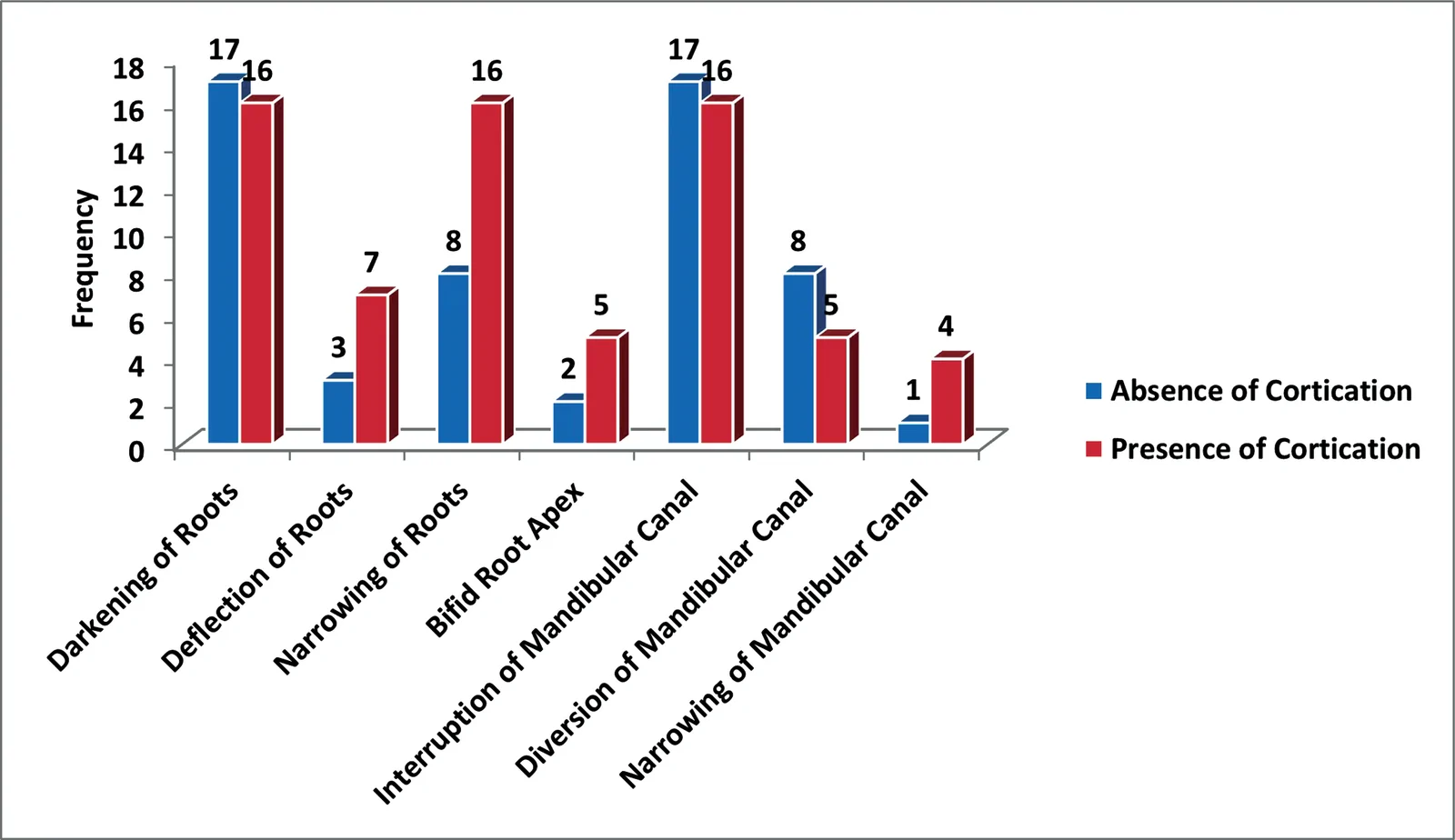
Graph representing Rood and Shehab’s radiographic signs with the presence and absence of cortication.

Table **3** among the radiographic signs of Rood and Shebab’s in CBCT. Highly significant values were obtained for darkening of roots (*p*<0.001), interruption of mandibular canal (*p*<0.001) and significant (*p*<0.04) for diversion of mandibular canal. Accuracy was highest for diversion of mandibular canal (70%), followed by darkening of roots and interruption of mandibular canal which was 66% respectively. The sensitivity of 94.4% was obtained for both the parameters (darkening of roots and interruption of the mandibular canal). All the other parameters were deemed to be statistically insignificant. A kappa value of 0.38, which is known to be fair agreement, along with sensitivity of 94.4%, specificity of 50%, PPV of 51.5%, NPV of 94.1% and an accuracy of 66% was obtained for darkening of roots along with interruption of mandibular canal. Likewise, diversion of mandibular canal, Kappa value of 0.31, which was fair agreement, with sensitivity of 44.4%, specificity of 84.4%, PPV of 61.5%, NPV of 72.9% and accuracy of 70% was determined (Table **3**, Fig. **6**).

## 4 DISCUSSION

Radiology plays a crucial role in the diagnosis of diseases prior to treatment. The primary objective is to identify morphological structures and their anatomical location surrounding the vital structures. Issrani *et al*. [14] noted that the risk of nerve injury following mandibular third molar surgery ranges from 0.6–5.3%, with the risk of permanent IAN damage remaining below 1%. This study included the Winter’s classification for evaluating the impacted mandibular third molars [4]. Similarly, research done by Passi *et al*. [15] and Jeevitha *et al*. [3] demonstrated that paraesthesia most commonly occurred with mesioangular and horizontal impactions, respectively. These findings were confirmed in our study, showing a lack of cortication between the IAC and impacted third molar with statistical significance (*p* <0.01) in horizontal impaction. Mesioangular impaction cases also showed contact between the impacted third molar and inferior alveolar canal with absence of cortication with significance (*p*<0.05), highlighting the importance of preliminary evaluation in these cases.

The efficacy of successful therapy depends on patient outcomes and treatment planning, making evidence-based selection of appropriate radiographic techniques vital. Before the use of CBCT, OPG was considered an ideal modality for evaluating the impacted teeth. Studies by Rajasekar *et al*. [16], Talib *et al*. [17], and Al-Haj Husain *et al*. [18] showed that OPG linear measurements influenced classification for predicting treatment outcome complexity. However, OPG provides less accurate data due to its two-dimensional limitation and inability to capture dental arch contours accurately because of image distortion [8]. Jaju *et al*. [19] emphasized that the main advantage of CBCT lies in its three-dimensional imaging capability, particularly useful for examining impacted teeth, while maintaining radiation exposure principles of ALARA (As Low As Reasonably Achievable), now modified to ALADA (As Low as Diagnostically Achievable). Sun *et al*. [20] demonstrated that CBCT assessment of lower impacted third molar complexity has become the ultimate module for treatment planning to avoid potential complications. According to Shaker *et al*. [21], it is a preferred diagnostic approach for clinicians evaluating mandibular third molars. In this study, all the subjects who had impaction demonstrated at least one Rood and Shehab’s [7] radiographic sign when evaluated with CBCT. Subsequent comparison of the presence or absence of cortication was assessed to estimate the risk of paresthesia. Hung *et al*. [22] found that cortical thinning was most frequently associated with lingual canal position relative to mandibular third molar roots. These findings were in accordance with our study, which showed a significant absence of cortication between the IAC and impacted third molar roots on the lingual side with high statistical significance (*p*<0.001), while the buccal side showed no significance. Cases with no cortication in the interradicular and inferior IAC positions showed no statistical significance. Studies conducted by Putrino *et al*. [23] and Yamada *et al*. [24] reported neurological complication rates of 0.5–1% for permanent damage and 5–7% for temporary damage, emphasizing the increased risk between impacted mandibular teeth and the IAC canal. This necessitates the need for accurate radiographic topographic diagnosis to understand root morphology and its relationship with vital structures.

Multiple studies have emphasized the critical relationship between mandibular third molars and IAC, indicating potential post-extraction complications. Ali *et al*. [25] evaluated 200 patients with impacted mandibular third molars and confirmed that root darkening is the predominant panoramic sign when comparing OPG and CBCT. Similarly, Jaron *et al*. [26] conducted a comparative study between OPG and CBCT, confirming differences in root darkening. Ruiz-Roca *et al*. [27] also demonstrated that root darkening played a prominent role in determining the risk of paraesthesia, which is in accordance with our findings where 33 patients exhibited root darkening, in which 16 cases had cortication between the impacted third molar and IAC, and 17 cases showed absence. The difference demonstrated high statistical significance (*p*<0.001), highlighting the importance of root darkening in OPG for potential nerve injury risk and emphasizing the necessity of CBCT before extraction.

Rizqiawan *et al*. [28] reported healing complications in patients with deflected mandibular root impactions. Del Lhano *et al*. [29] determined that while panoramic radiography effectively identifies the risk of IAN injury, CBCT support remains essential. They found that OPG radiographic indices, including root darkening and white line interruption, aided in evaluating the risk of nerve injury. Root deflection proved to be unimportant, which supports our study, where among 10 cases with root deflection, 7 showed the presence of cortication and 3 showed absence, without statistical significance.

A prospective study done by Kim *et al*. [30] identified that distoangular impaction causes more damage to the IAN during extraction. Narrowing of the roots of impacted mandibular third molars was also frequently associated with paraesthesia. Likewise, Edgar *et al*. [31] documented that intimate contact between the roots of impacted third molars and the inferior alveolar canal (IAC), particularly when accompanied by root narrowing, increases the risk of paraesthesia up to 11.1%, with 0.4% risk of permanent damage. It was observed that narrowing of the roots plays a significant role in assessing the risk of paraesthesia, although no statistical significance was found. These findings are similar to our study, in which 8 cases with narrowing of the roots of impacted mandibular third molars had no bone demarcation between the tooth and IAC, while 16 cases had cortication between the two structures, with no statistical significance. In a study done by Shashank Patel *et al*. [2] involving 120 individuals who had undergone both OPG and CBCT, the authors concluded that Rood and Shehab’s [7] radiographic signs are valuable in preventing potential complications during third molar extraction. Similarly, Shokri *et al*. [32] reported that CBCT provides more accurate information regarding the relationship between impacted mandibular third molars and the IAC. They noted that canal deviation and darkening of the root apex were associated with the highest risk of postoperative complications. This is consistent with our findings, in which a total of 7 cases with bifid root apices were observed, of which 5 confirmed contact between the roots and the canal, and 2 without contact. In our study, a clear interruption of the mandibular canal was noted in 33 patients: 17 without contact with the canal and 16 with cortication between the structures with high statistical significance (*p*<0.001). The incidence was lower than that reported by Tassoker *et al*. [33], who concluded that interruption of the mandibular canal increases the risk of nerve injury. In another prospective study, Waseem *et al*. [34] examined 956 OPG images containing 3,499 third molars and found that interruption of the mandibular canal was significantly associated with paraesthesia. Mendonca *et al*. [35] compared OPG and CBCT in evaluating mandibular canal interruption in patients undergoing third molar extraction, highlighting its role in assessing nerve injury risk which was quite evident in our study.

Diversion of the canal was observed in 37 cases in our study, with 10 cases showing clear cortication between the impacted mandibular third molar and IAC, and the other 27 lacking bone demarcation, and this difference was statistically significant (*p*<0.04). Meller *et al*. [36] compared OPG and CBCT scans, in 443 patients and found that canal diversion is an important predictor of post-extraction trauma risk. Izzetti *et al*. [37] concluded that diversion of the mandibular canal carries a 12-fold higher risk of IAN injury during surgical extraction, which aligns with our findings. Similarly, Kim *et al*. [30], concluded that canal diversion is a significant factor in predicting paraesthesia risk when they examined 650 cases.

In our study, narrowing of the mandibular canal was observed in 5 cases: 4 with cortication and 1 without and there was no statistical significance. Likewise, Koskela *et al*. [38] explained that alterations in the canal wall can be a key factor contributing to patient morbidity during the extraction of impacted mandibular third molars. Rieder *et al*. [39] documented that mandibular canal deviation can influence the rate of neurosensory impairment following surgical extraction. Mubarak *et al*. [40] reported that CBCT offers a more accurate assessment of surgical difficulty in third molar extractions than OPG. Ahamed *et al*. [41] in a study with 142 impacted mandibular teeth, compared OPG and CBCT in evaluating neurovascular bundle exposure and concluded that CBCT is valuable in assessing injury risk to the neurovascular bundle. This aligns with our findings, as we compared OPG images with CBCT and determined that CBCT is essential in estimating the risk of IAN injury to prevent serious complications.

In a study done by Sorooshzadeh *et al*. [42] possible causative factors affecting the complexity of third molar surgery in 526 patients were analysed with CBCT, where 345 cases (65.6%) had contact with roots of third molar and IAN, of which 180 patients were having horizontal impaction and 165 patients had mesioangular impaction associated with lingual position of canal, which is evident with our study, in which 11 patients had horizontal impaction and 14 with mesioangular impaction. Highly statistically significant results were obtained with the lingual position of the canal, indicating the presence of contact between the roots of the mandibular third molar and the mandibular canal.

Likewise in another study done by Horatiu Urechescu *et al*. [43] 253 CBCT scans of fully developed lower third molars were evaluated to check the position of IAC at lingual & interradicular positions, where (class III &class IV) were at high risk for potential nerve damage during surgical extraction of mandibular third molar. Similarly, in our study, there was a lack of cortication between the roots of lower third molars and IAN, when the position of IAC was lingual to the ridge of mandibular cortical bone.

Another study conducted by Khan *et al*. [44], two-arm parallel research comparing OPG & CBCT with variables like angulation, depth, difficulty index, Rood & Shehab’s criteria, they concluded that OPG alone cannot predict the risk of nerve damage even though the procedure was performed by an experienced surgeon, especially when Rood & Shehab’s findings seen in OPG were correlated with CBCT. It was known that there was a lack of cortication between the roots of the mandibular third molar and IAC indicating the risk of damage to IAN. This was evident in our study, where Rood & Shehab’s signs, such as darkening of roots, interruption of the mandibular canal were highly significant (*p*<0.001), indicating that CBCT acts as a valuable tool in preventing the damage of the mandibular canal during the surgical extraction of lower third molars.

In a systematic review done by Leung *et al*. [45] they advocated that traditional plain radiographs like OPG have been used for routine purposes, and are not accurate in predicting the risk of IAN injury while performing surgical extraction procedure for mandibular lower third molar, however with the advanced imaging techniques like CBCT, more information through 3D imaging and proximity of tooth root to mandibular canal can be clearly navigated through slices, which was clearly seen in our study, where presence of Rood & Shehabs radiographic signs were evident and were better visualized through CBCT .

In another systematic review by Shokri *et al*. [32] in all 4 databases PubMed, Scopus, Web of science and Google scholar from 1985-2024 it was ruled out that higher prevalence of paraesthesia is seen in roots darkening of Rood & Shehab’s radiographic sign and CBCT is highly efficient in detecting this sign which was in accordance to our study and statistical high significance of (*p*<0.001) was obtained.

In another study conducted by Sivakumar *et al*. [46] in 254 individuals with impacted mandibular third molar, the parameters like mandibular height, angulation of second molar and placement of canal using OPG and CBCT were evaluated, where they concluded that CBCT is much more effective diagnostic tool to rule out the presence of contact between roots of mandibular third molar and mandibular canal .This study was in accordance with our findings in which all the patients were assessed for OPG and CBCT, where CBCT was the gold standard in diagnosing the association between roots of lower third molars and IAN.

## 5 LIMITATIONS OF THE STUDY

 In the current study the sample size was small, with a limited geographical distribution of the sample attributed to the selected subjects which was considered a major constraint. However larger sample size and a wider geographical area could fetch conclusive results in the future. Also, the protocol of CBCT is mandatory for patients undergoing mandibular third molar extraction, not all patients can afford to take it, as CBCT is expensive, and expertise is needed to study the radiograph precisely, which may not be possible with general dentists. Post-operative clinical findings must be correlated with Rood and Shehab’s findings for the interruption of the mandibular canal, which may still add accuracy to the current study. Also, Radiovisography (RVG) a form of intraoral radiography which is known to be a useful diagnostic module was not considered in this study, which is known to be a major restraint.

## 6 FUTURE PROSPECTS

 The CBCT imaging of the mandibular third molar should be applied when the surgeon is in a dilemma and has a specific clinical question that can differ in individual patient cases, where OPG cannot solve the problem. Clear-cut evaluation using the latest diagnostic modules, like Cone Beam computed tomography, can minimise the risk by prior evaluation and careful planning of the surgical procedure. Current article concludes a valuable protocol to the oral surgeons, that CBCT must be made mandatory and an ideal guideline in the surgical procedure for impacted mandibular third molars. The prospect on low dose CBCT along with cost effective protocol can help in reducing post-operative complications and reducing morbidity.

## CONCLUSION

 To conclude, in the current study, CBCT was considered as a gold standard, and diagnostic accuracy was higher than OPG. There was fair agreement about the mesioangular and horizontal impaction and their proximity to the mandibular nerve. Likewise, among the radiographic signs of Rood and Shehab’s darkening of roots, interruption of the mandibular canal, and diversion of the mandibular canal were more accurately seen in CBCT and a sensitivity of 94% was obtained. CBCT is therefore essential for oral surgeons in evaluating the risk of nerve damage during extractions, with lingual positioning being more prone to damage, leading to paraesthesia. Careful evaluation and thorough investigations using CBCT can significantly reduce morbidity associated with the surgical extraction of mandibular third molars.

## Figures and Tables

**Table 1:** Comparison of position of the tooth according to Winter's classification of impacted mandibular third molar with presence or absence of cortication in CBCT.

**Tooth Position**	**Presence of Cortication**	**Absence of Cortication**	**Total Case**	***p*-value**
**Vertical**	9	6	15	0.07
**Horizontal**	2	9	11	**0.01**
**Mesioangular**	8	6	14	**0.05**
**Distoangular**	6	4	10	0.06

**Table 2:** The position of IAC with presence or absence of cortication in CBCT.

**Position of IAC**	**Presence of Cortication**	**Absence of Cortication**	**Total Case**	***p*-value**
Lingual side	5	9	14	**0.001**
Inferior	18	5	23	0.07
Inter Radicular	3	1	4	0.09
Buccal	7	2	9	0.07

**Table 3:** Comparison of Rood and Shehab's radiographic signs seen in CBCT with presence or absence of cortication using Kappa statistics.

**Rood & Shehab’s Radiographic Sign**	**Indication**	**Cortication Absent**	**Cortication Present**	**Total**	***p*-value**	**Kappa Value**	**Sensitivity**	**Specificity**	**PPV**	**NPV**	**Accuracy**
**Darkening of Roots**	Yes	17	16	33	**0.001**	0.38	94.4%	50%	51.5%	94.1%	66%
	No	1	16	17							
**Deflection of Roots**	Yes	3	7	10	0.7	-0.06	16.7%	78.1%	30%	62.5%	56%
	No	15	25	40							
**Narrowing of Roots**	Yes	8	16	24	0.8	-0.05	44.4%	50%	33.3%	61.5	48%
	No	10	16	26							
**Bifid Root Apex**	Yes	2	5	7	1	-0.05	11.1%	28.6%	84.3%	62.8%	58%
	No	16	27	43							
**Interruption of Mandibular Canal**	Yes	17	16	33	**0.001**	0.38	94.4%	50%	51.5%	94%	66%
	No	1	16	17							
**Diversion of Mandibular Canal**	Yes	27	10	37	**0.04**	0.31	44.4%	84.4%	61.5%	72.9%	70%
	No	5	8	13							
**Narrowing of Mandibular Canal**	Yes	1	4	5	0.6	-0.08	5.6%	87.5%	20%	62.2%	58%
	No	17	28	45							

## Data Availability

The data of current study are available from corresponding author, [A.A], on a reasonable request.

## LIST OF ABBREVIATIONS

RVG: Radiovisography
CBCT: Cone beam computed tomography
IAN: Inferior alveolar nerve
OPG: Orthopantomogram
SAGER: Sex and Gender Equity in Research
